# Intention Detection Using Physical Sensors and Electromyogram for a Single Leg Knee Exoskeleton

**DOI:** 10.3390/s19204447

**Published:** 2019-10-14

**Authors:** Dae-Hoon Moon, Donghan Kim, Young-Dae Hong

**Affiliations:** 1 Department of Electrical and Computer Engineering, Ajou University, Suwon 16499, Korea; anseogns56@ajou.ac.kr; 2 Department of Electrical Engineering, Kyung Hee University, Yongin 17104, Korea; donghani@khu.ac.kr

**Keywords:** knee exoskeleton, self-alignment, neural network, intention detection, LBKA sensor, electromyogram (EMG), sensor fusion

## Abstract

In this paper, we present a knee exoskeleton. Due to the complicated structure of the knee, an exoskeleton can limit the wearer’s movement (e.g., when completely sitting down). To prevent this, the proposed exoskeleton is designed to move the ankle part prismatically, so the movement of the wearer is not limited. In addition, the developed exoskeleton could be worn on only one leg, but in this case, it is difficult to detect the intention because the relative relationship information of the two legs is unknown. For this purpose, the length between the knee center of rotation and the ankle (LBKA) was measured and used for intention detection. Using a physical sensor—an encoder and an LBKA sensor, the success rate of intention detection was 82.1%. By additionally using an electromyogram (EMG) sensor, the success rate of intention detection was increased to 92%, and the intention detection was also 27.1 ms faster on average.

## 1. Introduction

The development of medical technology has increased the lifespan of human beings, and as a result, the number of people who feel discomfort when walking due to the aging of joints is increasing. To solve this inconvenience, studies are underway on exoskeletons that assist the joints. In this paper, we present a knee exoskeleton because the knee is vulnerable to wear aging, which is difficult to treat. In addition, the knee is a joint frequently used in real life and deals with a lot of loads. 

The knee has a complicated structure, so it is difficult to use an exoskeleton on knee [1]. As a result of this structure, the center of rotation of the knee changes with the movement of the knee. If the exoskeleton is designed without considering the change of the center of rotation, the range of motion of the user is limited. To solve these problems, exoskeletons considering complex knee movements have been studied. Studies have been carried out that use a link system to self-align [2] and that use Schmidt coupling to accommodate large radial displacements between two shafts [3]. However, such exoskeletons have a disadvantage in that the volume of the actuator is large. There are the exoskeletons that have a prismatic degree of freedom to self-align with the complex movements of the knee [4,5,6]. Due to this structure, the exoskeleton does not limit the wearer’s range of motion; moreover, there is no need to enlarge the volume of the actuator. The difference between [4,5,6] and the exoskeleton suggested in this paper is that the proposed exoskeleton has the structure which measures the change of the center of rotation. Though the change of the center of rotation is measured in [6], they use the displacement sensor, which is attached to the tibia model. However, the proposed exoskeleton in this paper could be equipped to the actual wearer because the sensor is attached inside the exoskeleton. In this paper, the movement of the center of rotation is measured and used to detect intention. As a result, the intention is detected by using the data obtained by only one leg.

An exoskeleton worn on one leg, such as a single leg version of Hybrid Assistive Limb (HAL), is studied in [7]. A person with discomfort in only one leg needs an exoskeleton to be worn only on that leg. However, since an exoskeleton worn on one leg, information about the relative relationship between both legs cannot be known, so it is difficult to detect the intention of the wearer. In this study, this problem is solved by using various sensors.

Many sensors have been used for intention detection. An encoder is used to measure the angle of the joint [8]. Electromyogram (EMG) measures the action potential transferred from the motor neurons to the muscle fibers [9]. Therefore, EMG is used to obtain motion data before the actual motion. Moreover, a new kind of sensor has been developed, which estimates the torque by sensing the movement of the muscles using air pressure [10]. Studies have also been conducted in the aspect of sensor fusions. EMG and encoder sensors are fused in [11] to develop an EMG-based impedance control method to control a robot in accordance with the user’s motion intention. In the proposed exoskeleton, the encoder value and the length between the knee center of rotation and the ankle (LBKA) caused by the movement of the knee are measured and used for intention detection. The LBKA sensor is important because it can detect the inside movement of the knee joint. By measuring the LBKA, accurate intention detection is possible while using fewer sensors, and the encoder delay problem caused by the wire driven actuator is solved. In addition, to increase the performance of intention detection, EMG is fused with the two sensors.

There are many ways to detect intention. Simple heuristic threshold methods have been used previously [12]. Although their implementation is simple, they have a disadvantage in that the user must manually determine the threshold. Fuzzy models detect intention using a base rule [13]. An engineer can create a rule base, but it is tricky. Studies have also used the mathematical maximum value problem of support vector machines [14]. However, this approach faces the difficulties of manually selecting many variables. In this study, intention detection is performed by using a neural network (NN). A NN expresses neurons mathematically and detects intention using these neurons.

In this study, we designed an exoskeleton that supports only one knee with a self-alignment structure. Furthermore, the exoskeleton is used to detect the intention of the stair ascending motion. Because it uses data from only one leg, it is difficult to detect the intention. To solve this problem, we used LBKA, encoder, and EMG data to ensure that intention detection had a high success rate and reliability.

The composition of the paper is as follows. Section 2 contains a description of the anatomical motion of the knee. In Section 3, we elaborate on exoskeleton design for self-alignment and the role of the ankle variable section. Section 4 provides an explanation of the EMG data acquiring method and neural network. In Section 5, we describe our experimental results, and conclusions follow in Section 6.

## 2. The Anatomical Motion of the Knee

**Figure 1:**
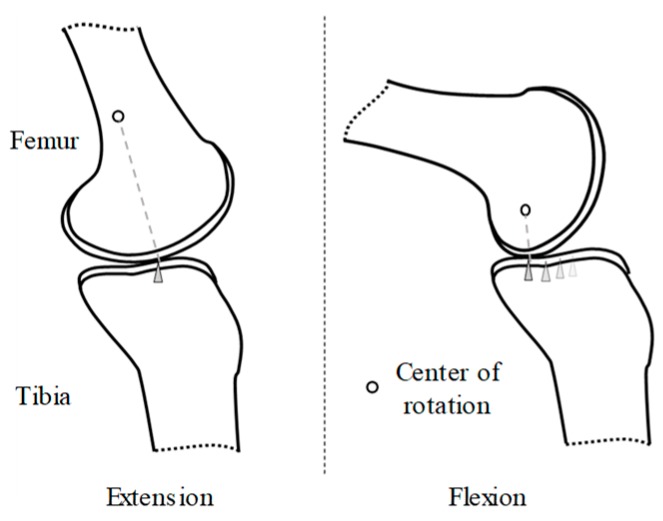
Movement of the femur on the tibia plane. The contact point and center of rotation change according to the movement of the knee.

Figure 1 shows a knee composed of a femur, tibia, and extra-articular structures. Since the movement of the knee is complex, it is represented by six degrees of freedom (DoF) [15]. However, extension and flexion account for most of the movements of the knee joint. The movement of the knee is made by moving the egg-shaped femur back and forth on the flat tibia plane. Due to the contact point movement and the femur and tibia bone shape, the center of rotation of the knee changes as the knee moves, and the LBKA is changed. For this change, the proposed exoskeleton is designed to align the center of rotation of the exoskeleton with the center of rotation of the wearer. These structures are explained in Section 3.

## 3. Exoskeleton Design for Self-Alignment

### 3.1. Overall Exoskeleton

**Figure 2:**
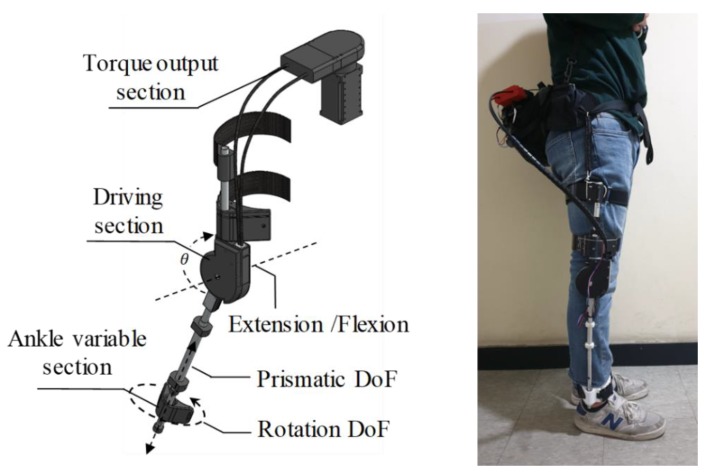
The overall appearance (**left**) and actual appearance (**right**) of the exoskeleton. The exoskeleton has three DoFs: two on the ankle and one on the knee. The knee angle is given as *θ*. The lower frame length of the exoskeleton could be changed so that the center axis of the knee rotation and the axis of rotation of the exoskeleton could be matched. Thus, fully seated motion is possible.

Figure 2 shows the overall appearance of the knee exoskeleton. This exoskeleton has three DoFs. There is an active DoF connected to the motor on the knee. The angle measured by the encoder of the motor is expressed as *θ*. Passive prismatic and passive rotation DoFs in the ankle allow the rotational center axis of the exoskeleton to align with the rotational center axis of the knee and are used to measure LBKA.

The exoskeleton is designed in three parts: the torque output section, the driving section, and the ankle variable section. The torque output section has heavy parts such as batteries and motors and is worn on the wearer’s back. If the heavy parts are directly worn on the knee, the weight could cause the wearer to feel a lot of inertia force when moving the knee. The torque generated by the motor located in the torque output section is transmitted to the driving section using Bowden cables.

The driving section applies the torque generated in the torque output section on the knee. Because the driving section is worn on the knee, its weight is important to reduce the inertia force. Therefore, the heavy parts are placed in the torque output section, and the driving section is made of light ABS material and an aluminum frame to make it hard and light. By this design, the weight of the driving parts does not exceed 800 grams.

The operating range of the exoskeleton is 145 degrees, which is beyond the human knee range of 140 degrees, so the wearer’s range of motion is not limited by the exoskeleton [16].

### 3.2. Ankle Variable Section Design for Self-Alignment

**Figure 3:**
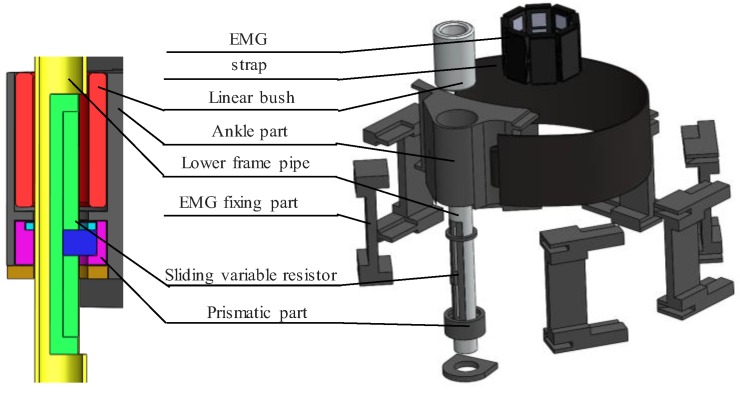
Exploded diagram to explain the ankle variable section.

Figure 3 shows an exploded view of the ankle section. Because the LBKA changes with the movement of the knee, the length of the lower exoskeleton frame should change. For this purpose, the ankle part freely moves prismatically along the lower frame pipe and rotates because the linear bush eliminates the friction between the lower frame pipe and the ankle part. 

LBKA changes with knee movement. Therefore, it is possible to detect human intention by measuring the change in LBKA. A sliding variable resistor is installed in the lower frame pipe to measure changes in LBKA; it can only move prismatically, but the ankle part can rotate freely along the lower frame pipe. For this reason, if you connect the sliding variable resistor directly to the ankle part, its prismatic movement friction will rise due to the rotation of the ankle part. The LBKA value cannot be accurately measured because of this friction. To prevent this friction, the prismatic part is connected to a sliding variable resistor to move prismatically along the lower frame pipe. The ankle part is then connected to the prismatic part and rotates around it, so the rotation of the ankle part does not cause friction in the sliding variable resistor.

To fix the EMG to the ankle, the EMG fixing part is connected to the strap. Through this structure, the EMG is always attached in a similar position, and so a similar EMG value is measured when initializing the EMG. This part also prevents the ankle strap from sagging, so that the LBKA measurement becomes more accurate.

**Figure 4:**
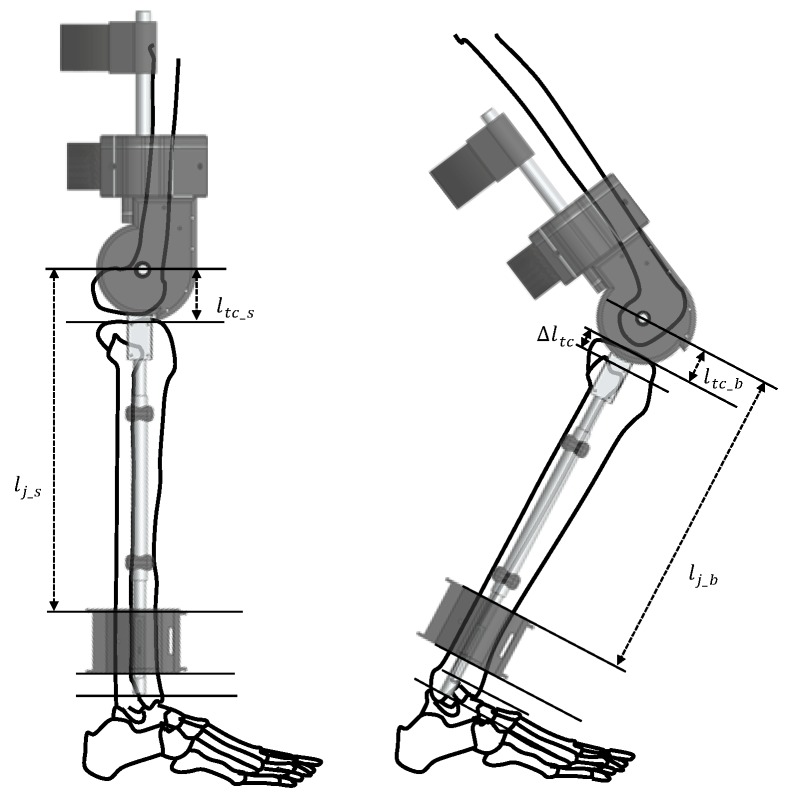
Movement of ankle variable section while moving the knee. As the knee flexes, the LBKA becomes shorter. As a result, the lower frame length of the exoskeleton becomes shorter.

Figure 4 illustrates the movement of the ankle variable section while moving the knee. This could be expressed as

(1) $$l_{tc\_b}=l_{tc\_s}-\Delta l_{tc}$$

The distance between the center of rotation of the knee and the tibia plane $l_{tc}$, changes with the movement of the knee. The $l_{tc}$ value is $l_{tc\_s}$ when the knee is stretched, but it is decreased by $\Delta l_{tc}$ when the knee is bent, so it becomes $l_{tc\_b}$. Therefore, the length of the lower frame is $l_{j\_s}$ when the knee is stretched changes to $l_{j\_b}$ when the knee is bent. By using the ankle variable section movement, the axis of rotation of the exoskeleton is aligned with the change in the knee center axis. With this design, the load felt by the wearer due to the mismatch of the axis is reduced, and a completely sitting motion is possible. This is shown experimentally in Section 5.1.

The encoder is attached to the motor that is connected to the knee joint by Bowden cable, so the encoder measures the angle information of the knee. However, the motor is not directly attached to the knee, so the knee angle measured by the encoder of the motor is delayed information. On the other hand, the LBKA changes without delay because it changes according to the internal motion of the knee joint. In addition, because the ankle sensor measures the movement of the joints inside, it reflects the intention of the wearer well. Therefore, intention detection is possible with only two sensors of one leg.

## 4. Acquiring EMG Data and Neural Network

### 4.1. EMG Filtering

EMG measures the action potential of the muscle fibers induced by the action potential of the motor neurons. Because one motor neuron dominates multiple muscle fibers, the action potential of the multiple muscle fibers is measured simultaneously through the electrodes. The measured EMG is determined by various factors, such as the rate of activation of the action potential and the number of muscle fibers measured on the electrodes. The EMG value is faster than the physical sensors because the EMG measures the value of the action potential delivered to move a muscle.

The EMG data are integrated for filtering because of the value obtained by integrating correlates with the muscle contraction force of the wearer [17]. The noise of the EMG is also removed through this filtering. The filtering process is expressed as

(2) $$\mathrm{iEMG}=\sum_{n=1}^{N}\frac{\left|V\left(n\right)\right|}{N},\;\left\{n=1,2,3\ldots \;N\right\}.$$

### 4.2. The Neural Network for Intention Detection

**Figure 5:**
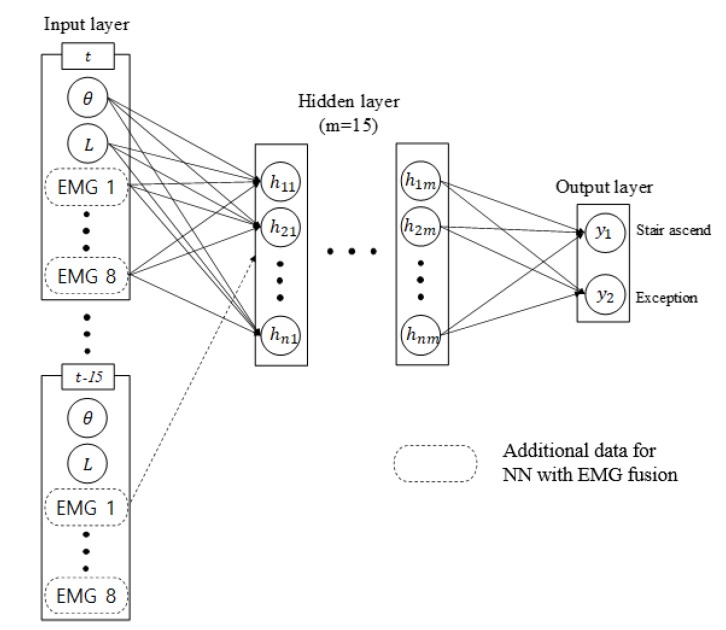
Structure of the NN with a physical sensor only and the NN with EMG fusion. Both NNs use the encoder and the LBKA values as input, but the NN with EMG fusion additionally uses EMG data. The output of the NN is the probability of the stair ascending and exception states.

Figure 5 illustrates the structure of the NN trained by two different sources of input. The Tan–Sigmoid activation function is used as the neuron model in the hidden layer, and the soft-max activation function is used in the output layer. The network is trained with scaled conjugate gradient backpropagation. Both NNs are trained with LBKA and the encoder values as input, but the NN with EMG fusion additionally uses filtered EMG data. Every sensor value is then normalized because the data of sensor that has small values are ignored in the training process. The NN is trained for two states—stair ascending is the moment at which the wearer begins to extend the knee to ascend the stairs, and all other moments are trained as exceptions.

**Figure 6:**
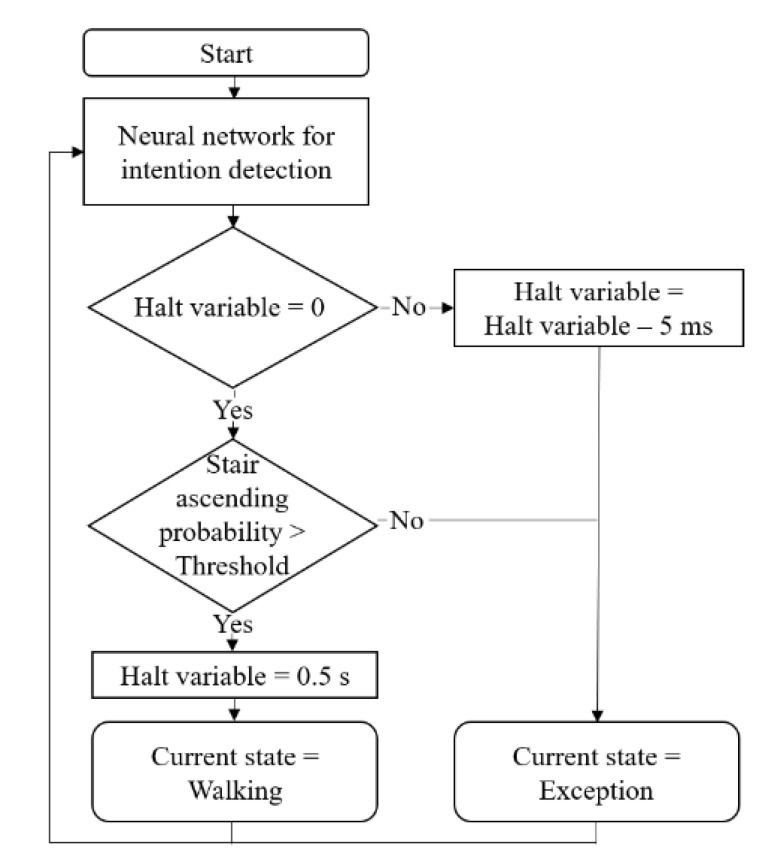
Algorithm for the state decision. If the probability of the stair ascending state output by the NN exceeds the threshold, change the current state to stair ascending. After that, the current state becomes the exception state for 0.5 s, so that one stair ascending action is not detected as multiple stair ascending actions.

Figure 6 shows the algorithm for determining the current state based on the probability output of the NN. This output does not indicate a definite state, so it cannot be used in practical uses such as torque generation. Therefore, an algorithm that clearly determines the current state is needed. If the probability of the stair ascending state exceeds the threshold, the current state is determined as stair ascending. Otherwise, the current state is determined as the exception. The exception state is every moment that is not intended to ascend stairs. Threshold values are determined by examining the output probability of the trained NN. Firstly, threshold values are set higher than the output probability of the trained NN when there is no actual intention, so that no false intentions would be detected. Secondly, the threshold values are set as low as possible. The reason is that if threshold values are set lower, the intention can be detected even though the output probability of the trained NN is low when there is the actual intention. Therefore, the lower the threshold values are set, the faster the state decision algorithm responds to the output probability of the trained NN, allowing for faster intention detection. Considering these two factors, the threshold values are determined.

We also added the halt variable not to detect one stair ascending action as multiple stairs ascending actions. The intention detection through NN is a method of pattern recognition, so the output value is not high only at the moment when the wearer’s actual intention occurs. There is a high probability even after the actual intention occurs because NN judges that there are similar patterns. To deal with these problems, the halt variable is set to 0.5 s when the current state is determined as a stair ascending state. After that, the algorithm subtracts 5 ms from the halt variable at each sampling time. The reason for subtracting 5 ms is that the sampling time of the entire system is 5 ms. The state decision algorithm sampling time is also 5 ms. Until the halt variable becomes zero, the current state is decided as an exception regardless of the probability of NN. In this way, detecting a stair ascending state is halted to for 0.5 s. Consequently, the intention is detected exactly at the moment of actual intention.

## 5. Experiment and Result

### 5.1. Range of Motion Experiment

**Figure 7:**
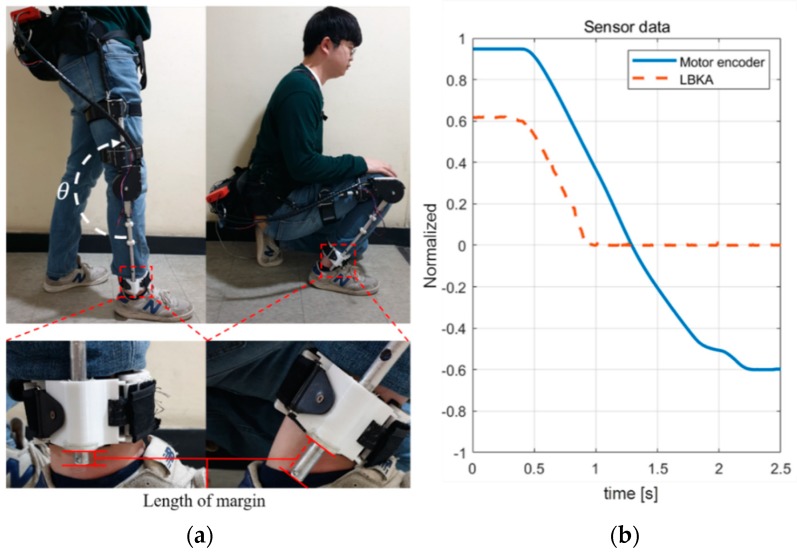
Range of motion experiment snapshot. As the knee is bent, the length of the lower frame of the exoskeleton decreases. This structure does not limit the wearer’s range of motion, so it is possible for the wearer to sit down fully. (**a**) Snapshot of the experiment and detailed view of the length of margin change in the ankle part. The angle measured by the encoder of the motor is expressed as *θ*, (**b**) Graph of measured data.

Figure 7 shows an experiment to confirm that the hardware does not limit the wearer’s movement. Data was measured when the wearer switched from a standing position to a fully seated position. The wearer weighed 82 kg and was 183 cm tall. And the sampling time of the entire system and the measured data was 5 ms. Three sensors are used for training: encoder, LBKA sensor, and EMG sensor. As described in Section 4.2, for smooth training, we normalized the measured sensor value. Normalization means rescaling data to have values between 0 and 1. The encoder value was rescaled to 0 when the knee was bent, so *θ* is 90 degrees and to 1 when the knee was extended, so *θ* is 180 degrees. The LBKA value was rescaled to 1 when the maximum change was made and to 0 when the change was minimum. The maximum change of the LBKA sensor is 4.0 cm, and the minimum change is 0.0 cm. In the case of the EMG sensor, the measured EMG value is rescaled to 1 when maximum and to 0 when minimum.

Figure 7a shows a picture of the beginning and the end of the experiment. As shown in the detailed view of the ankle part in Figure 7a, the length of the margin of the lower pipe changes as the length of the lower frame changes. As the wearer changes from a standing position to a sitting position, the length of the lower frame decreases, and the margin of the lower pipe increases. In addition, as shown in the Figure 7b graph, the measured LBKA value decreases as the knee is bent. These experiments show that the exoskeleton does not limit the wearer’s range of motion and that it enables a full sitting position.

### 5.2. Intention Detection Experimental Environment

**Figure 8:**
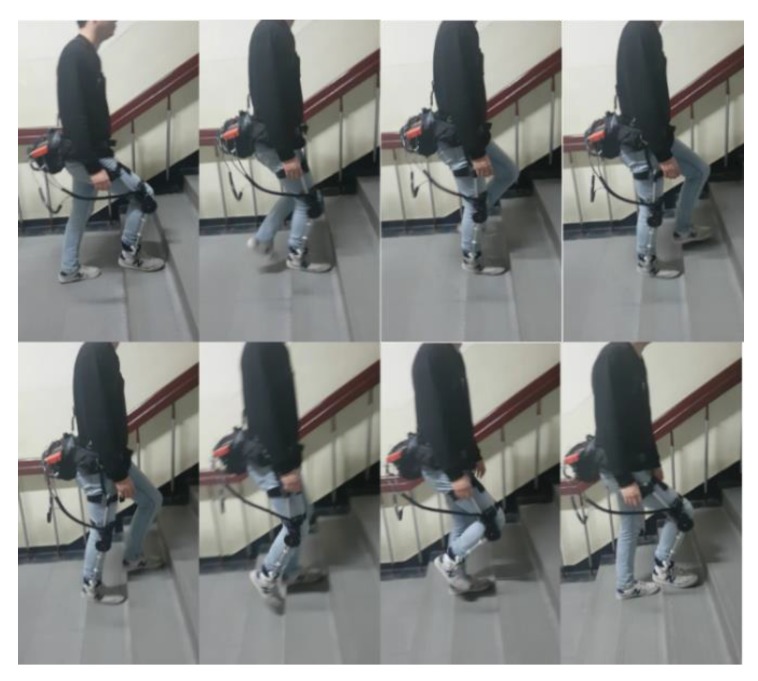
Stair ascending experiment snapshot. The trained NN was tested by ascending stairs while wearing the exoskeleton.

As shown in Figure 8, the person wearing the exoskeleton ascended the stairs continuously to generate data for the experiment. The collected data were used to train the NN and to test the trained NN. We analyzed two malfunction cases of NN with a physical sensor only, with and without actual intention. Through this analysis, we confirmed that the performance of NN with EMG fusion was better than the performance of NN with a physical sensor only. Subsequently, the difference between the success rate and the intention detection time of each NN was confirmed. We also measured the data when the wearer walked on the flatland to show that the trained intention detection algorithm does not malfunction with similar inputs.

The EMG used in the experiment had eight-channel electrodes. The EMG data, the encoder, and the LBKA values were collected by the program using a MATLAB GUI, which was also used to calculate the NN results.

### 5.3. The Change of the LBKA Sensor and Encoder Depending on Intention

**Figure 9:**
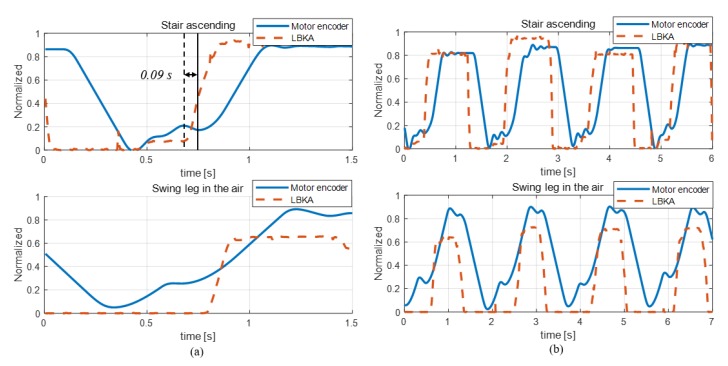
Graph of the actual ascending stair, and when the leg is swung in the air like the process of an ascending stair: (**a**) detailed motion view of each motion, (**b**) continuous motion data.

Figure 9 shows a graph of the data when actually ascending the stairs and when the leg is swung in the air like the process of stair ascending. In the stair ascending graph of Figure 9a, the vertical solid line indicates when the motor encoder is changed by the motion of the wearer, and the dashed vertical line indicates when the change of LBKA reacts to the motion of the wearer. The change of LBKA was faster than the knee angle measured by the encoder. The time value on the graph indicates the difference in time at which the two sensors begin to change due to the motion of wearer. As mentioned in Section 3.2, the LBKA sensor measures the change of LBKA caused by the movement of the internal joint, so there is no delay. However, as shown in the data of Figure 9b, the LKBA sensor values are different even when the encoder values are the same. Thus, the LBKA sensor does not have a delay but does not measure accurate angle values. On the contrary, the encoder has a delay but can measure the exact angle. Therefore, the intention is detected by the relationship between these two data. Also, as shown in Figure 9a, the encoder data shows that there are two slopes, both in the case of actual stair ascending and in the case of swing the legs in the air. However, the measured LBKA sensor data shows that there are two slopes in the case of stair ascending, but there is only one slope in the case of swinging the legs in the air. Consequentially, the knee movement measured by the encoder is the same in both cases, but the knee movement measured by the LBKA sensor is different. Due to the difference between the encoder and the LKBA sensor, the intention cannot be distinguished with the encoder of only one knee, but the intention can be accurately detected by the fusion of the two sensors.

### 5.4. Intention Detection Experimental Results

The NNs used for intention detection were implemented using the MATLAB toolbox. After heuristically experimenting with various hidden layers number, 15 hidden layers showed the best performance. Therefore, the number of hidden layers was determined to be 15. 70% of training data was used as a training set, 15% as a validation set, and 15% as a test set. The training set is data for training the NN, and the validation set is the data for preventing overfitting of the NN. The test set was used to verify that the training was successful after the NN training was completed. The stair ascending state used 20 training datasets, and the exception state used 140. The exception state required a lot of data because there were no fixed patterns. However, if the number of training set in the exception state (major state) was much larger than the stair ascending state (minor state), an error would occur in the training because of the imbalanced size of the data. If the data rate of the major state and minor state is 9:1, even if NN judges all cases as a major state, the overall success rate is 90%. However, the minor state has a success rate of 0%. This imbalanced dataset greatly affects the detection of minor states. In order to solve this problem, an error is prevented by using an under-sampling method that adjusts the number of major state data. 

**Figure 10:**
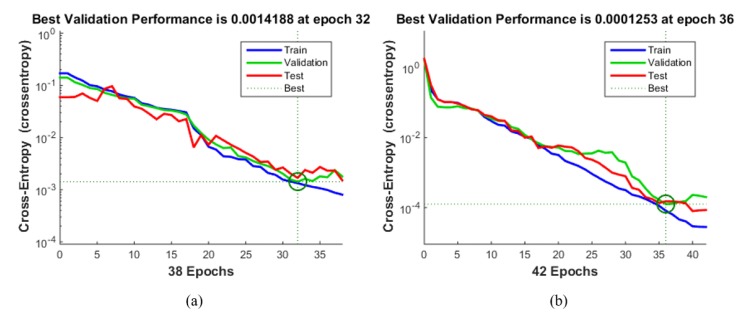
Cross entropy graph of NN according to epoch for the performance analysis: (**a**) NN with the physical sensor was only trained for 38 epochs, and (**b**) NN with EMG fusion was trained for 42 epochs.

Figure 10 shows the result of evaluating NN with a physical sensor only and NN with EMG fusion through cross-entropy. This is a stochastic calculation defined by the relationship between the estimated probability distribution and the true distribution. It is a typical loss function used when the output value of the NN is between 0 and 1. The error in the training set decreased after each chosen epoch, but the error of the test set increased. This means that overfitting occurs after the chosen epoch. Therefore, the training had been carried out correctly by selecting the epoch that had the best performance with both the test set and the training set.

The state decision algorithm thresholds of the NN with Physical sensor only and the NN with EMG fusion were set at 70% and 95%, respectively, by examining the probabilities calculated after training the NNs.

**Figure 11:**
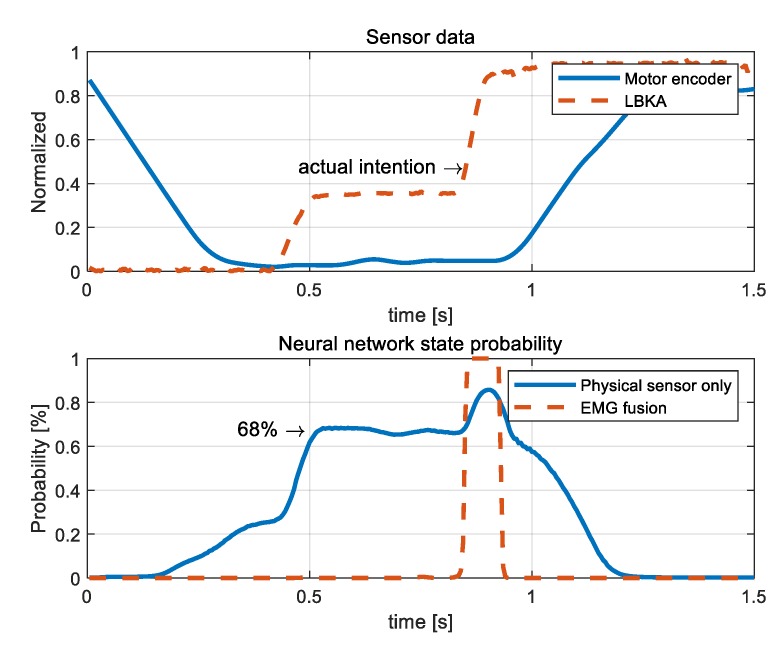
A graph of the measured sensor values and NN results. The probability of the NN with the physical sensor only was calculated as high even when there was no actual intention, whereas that of the NN with EMG fusion was calculated as high only when there was the actual intention.

Figure 11 shows that the NN with Physical sensor only malfunctioned when there was no actual intention. The values of the first graph in Figure 11 are the LBKA value and motor encoder value, and the actual intention is indicated in the graph. The second graph shows the probability output by the NNs. In the case of the NN with the physical sensor only, the probability was calculated as 68% even when there was no actual intention. At that moment, the wearer did not actually ascend the stairs, so the probability output by the NN should be low, but it was calculated as high. However, the NN with EMG fusion calculated the probability as 99% at the moment of actual intention.

**Figure 12:**
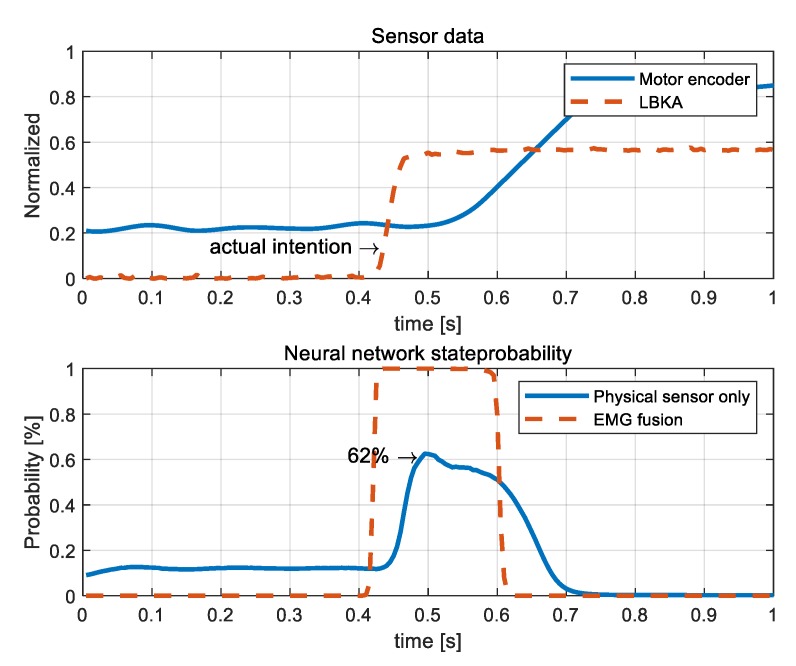
A graph of the measured sensor values and NN results. The probability output by the NN with the physical sensor was lower than even the malfunctioned case of Figure 11 when there was an actual intention. The probability of the NN with EMG fusion was calculated as high when there was an actual intention.

Figure 12 shows that the NN with the physical sensor only malfunctioned when there was a actual intention. The probability of NN with the physical sensor only is calculated as 62%. Since the wearer was actually moving, the probability should have been high, but it was calculated as lower than the probability of malfunction case in Figure 11. On the other hand, NN with EMG fusion calculated the probability to be 99% at the moment when the actual intention occurred.

The NN with Physical sensor only caused errors such that the probability was calculated as high even when there was no actual intention and was calculated as low when there was an actual intention. Therefore, the performance of the NN with the physical sensor only cannot be improved by adjusting the threshold of the state decision algorithm. In the case of the NN with EMG fusion, there is always over 99% probability when there is an actual intention. This analysis shows that the experimentally determined threshold of state decision algorithm is appropriate, and NN with EMG fusion is more robust than NN with the physical sensor only.

**Figure 13:**
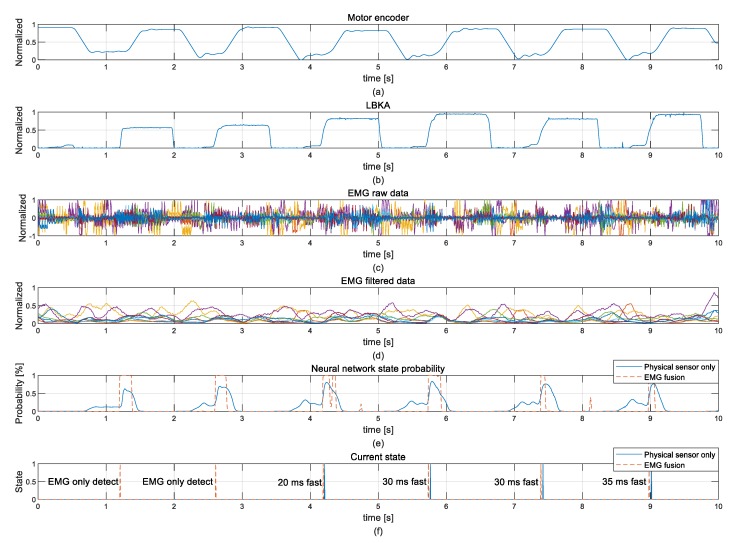
Experimental data for the continuous ascending stair: (**a**) motor encoder, (**b**) LBKA, (**c**) EMG raw data, (**d**) EMG filtered data, (**e**) NN probability, and (**f**) current state.

Figure 13 shows plots for 10 s of experiment data. The noise-intensive raw EMG data in Figure 13c became usable EMG data after using the integration filter, as shown in Figure 13d. Figure 13f shows the current state determined by the state decision algorithm. When comparing the current state decision times of each neural network, the current state is quickly determined in NN with EMG fusion in all cases. In the experiment, NN with EMG fusion averagely detected the stair was an ascending state 27.1 ms faster than the NN with the physical sensor only. In Figure 13f, only the NN with EMG fusion correctly detected the intention in the first and second stair ascending actions. Table 1 shows the detection rates of each NN. The detection rate of NN with EMG fusion (96.4%) was higher than the detection rate of the NN with the physical sensor only (82.1%).

**Figure 14:**
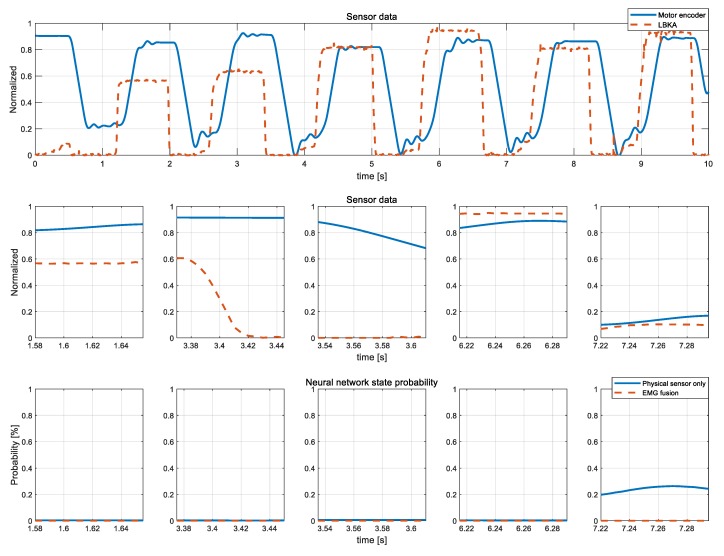
Detailed view of various cases of exception state and probability of each case.

Figure 14 shows the various patterns of the exception state from the stairs ascending experiment data in Figure 13. As shown in Figure 14, exception state is difficult to train because there is no fixed pattern. In each case, however, the NN calculates a probability of the ascending stair state close to zero. In this respect, NN is well trained about the exception state.

**Figure 15:**
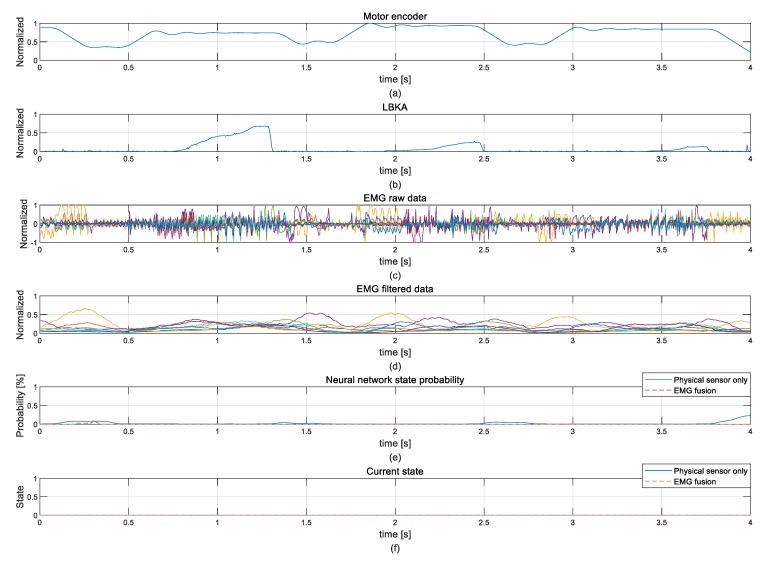
Walking level experimental data for exception state test: (**a**) motor encoder, (**b**) LBKA, (**c**) EMG raw data, (**d**) EMG filtered data, (**e**) NN probability, and (**f**) current state.

Figure 15 shows sensor data measured when the wearer walks on the flatland to see if the NN malfunctions when sensor data similar to the ascending stair is measured. The movement of the motor encoder when the level of walking is similar to the ascending stair, but NN does not detect false intentions. Since similar input values were not detected as false intentions, the trained NN is robust.

As a result, we verified that NN with EMG fusion detected intention more quickly and accurately than the NN with the physical sensor only. In addition, the threshold value of the state decision algorithm and the computed probability of NN proved that NN with EMG fusion is not only more successful at predicting intention but also more robust than the NN with the physical sensor only. Moreover, the example of the exception state and the similar input pattern experiment show that the trained NN is robust.

## 6. Conclusions

In this study, the single-leg knee exoskeleton was developed. Since knee movement is complicated, a self-alignment structure was applied to align the exoskeleton. Therefore, the exoskeleton does not limit the wearer’s range of motion, which makes it possible for the wearer to sit down completely. The LBKA value was measured, and used to detect intention using data from only one leg. Furthermore, the intention detection accuracy was increased by adding an EMG sensor and the intention detection became faster. In addition, the results of each NN were compared, which confirmed the higher reliability of using EMG and physical sensors together.

Our future work is to enhance the generality of intention detection. To enhance the generality, experiments will be conducted with several subjects while maintaining accuracy, even when the EMG value changes due to fatigue. Thereby, more training data can be obtained, and the intention detection algorithm can be tested on multiple subjects, so generality can be improved. For this purpose, skin attached EMG sensors are also in development.

## Figures and Tables

**Table 1:** Detectability factor of each NN.

Sensor	Physical Only	With EMG
Detectability factor	82.1%	96.4%
